# Hypervalent Iodine with Linear Chain at High Pressure

**DOI:** 10.1038/srep14393

**Published:** 2015-09-24

**Authors:** Shubo Wei, Jianyun Wang, Shiyu Deng, Shoutao Zhang, Quan Li

**Affiliations:** 1 State Key Laboratory of Superhard Materials, Jilin University, Changchun 130012, China; 2 College of Materials Science and Engineering, Jilin University, Changchun 130012, China

## Abstract

Iodine is an element of fascinating chemical complexity, and numerous hypervalent iodine compounds reveal vital value of applications in organic synthesis. Investigation of the synthesis and application of new type of hypervalent iodine compound has extremely significant meaning. Here, the formation of CsI_n_ (n > 1) compounds is predicted up to 200 GPa using an effective algorithm. The current results show that CsI_3_ with space group of *Pm*-3*n* is thermodynamically stable under high pressure. Hypervalence phenomenon of iodine atoms in *Pm*-3*n* CsI_3_ with endless linear chain type structure appears under high pressure, which is in sharp contrast to the conventional understanding. Our study further reveals that *Pm*-3*n* CsI_3_ is a metallic phase with several energy bands crossing Fermi-surface, and the pressure creates a peculiar reverse electron donation from iodine to cesium. The electron-phonon coupling calculations have proposed superconductive potential of the metallic *Pm*-3*n* CsI_3_ at 10 GPa which is much lower than that of CsI (180 GPa). Our findings represent a significant step toward the understanding of the behavior of iodine compounds at extreme conditions.

Iodine compounds have always been the subject of extensive studies because of their significant properties such as conduction characteristic^1^,^2^, optical property^3^, catalytic performance^4^,^5^ and medical application^6^, etc. For instance, cesium iodide (CsI) is one of the simplest and most representative ionic solids, and extensive theoretical and experimental studies have been carried out^7^,^8^,^9^,^10^,^11^,^12^,^13^,^14^,^15^. CsI exhibits variety of interesting phenomena such as pressure involved metallization and superconductivity under high pressure^12^,^13^,^14^. In addition, organic molecules bearing hypervalent iodine moieties have been transformed from laboratory curiosities to useful and routinely employed reagents in organic synthesis^16^,^17^,^18^,^19^,^20^,^21^. Comparing with the relatively and easily synthesized organic complexity, the synthesis of inorganic hypervalent iodine compounds with attractive properties and its corresponding structures have been a long-standing puzzle. For I_3_^−^ ion, its linear geometric structure was determined for the first time in 1935 by Mooney, who carried out X-ray analysis on ammonium triiodide^22^. The I_3_^−^ anion belongs to the type of compounds known as hypervalent, which violate the Lewis octet rule^23^. Due to the representative hypervalence property of I_3_^−^ ion, it is of great consideration for further study of CsI_n_ (n > 1) compounds to explore the possibility of forming hypervalent CsI_n_ with interesting properties or structures. Previous experimental works show that crystal CsI_3_ and CsI_4_ can be synthesized at ambient pressure^24^,^25^,^26^,^27^. Subsequently, experimental XRD measurement proposed that the crystal information of CsI_3_ and CsI_4_ were orthorhombic *Pmnb* and monoclinic *P*2_1_*/a* symmetry, respectively^28^,^29^,^30^. W. Zhang *et al*. did an excellent work for Na_x_Cl_y_ system by combining theoretical predictions and diamond anvil cell experiments^31^. They reported that Na_3_Cl, Na_2_Cl, Na_3_Cl_2_, NaCl_3_ and NaCl_7_ are theoretically stable and have unusual bonding and electronic properties at high pressure^31^. Na-Cl and Cs-I system are clearly analogous as typical ionic solids and they may adopt the same structures. Currently, the synthesis or the full high-pressure structural information of CsI_n_ are still far from being clear and established. These structural uncertainties have impeded in-depth understanding and further exploration of phenomena of CsI_n_ might under compression.

Here, we present systematic structure searches to establish the thermodynamically stable structures of CsI_n_ (n = 2 − 5) up to 200 GPa using the developed CALYPSO (Crystal structure AnaLYsis by Particle Swarm Optimization) method^32^,^33^,^34^,^35^, which has been successfully used in numerous predictions regarding compounds and structures over the past few years^36^,^37^,^38^,^39^,^40^,^41^,^42^,^43^,^44^,^45^,^46^. Our work shows that CsI_3_ has the simple *Pmnb* structure at ambient pressure and undergoes a complicated transition to high symmetric cubic *Pm*-3*n* phase at high pressure. The current *Pm*-3*n* phase of CsI_3_ is a metal phase, and the pressures create a peculiar reverse electron donation from iodine to cesium. In addition, the iodine atoms of *Pm*-3*n* phase forming into several completely linear chains show an attractive hypervalence phenomenon, which cannot be simply explained by traditional three-center-four-electron (3c-4e) scheme^47^,^48^,^49^. This work presents significant concerning on the synthesis and the fundamental structural properties of the simplest and most representative polyiodide materials with implications for an entire family of similar materials.

## Calculation Methods

To obtain stable structures for CsI_n_, we carried out a structural search using a global minimization of free energy surfaces based on the CALYPSO methodology^32^,^33^,^34^,^35^ and the first-principles calculations. The remarkable feature of this methodology is the capability of predicting the stable structure with only the knowledge of the chemical composition at given external conditions (for example, pressure). The underlying *ab initio* structural relaxations, electron localization function (ELF) and electronic band structure calculations were performed within the framework of density functional theory (DFT) as implemented using by VASP (Vienna *ab initio* simulation package) code^50^. The generalized gradient approximation (GGA) within the framework of Perdew–Burke–Ernzerhof (PBE)^51^ was used for the exchange-correlation functional. And the projector augmented wave method (PAW)^52^ has been adopted, with 5*s*^2^5*p*^6^6*s*^1^ and 5*s*^2^5*p*^5^ treated as valence electrons for cesium and iodine, respectively. The cutoff energy for the expansion of the wavefunction into plane waves was set at 400 eV and fine Monkhorst–Pack (MP) *k* meshes of 0.025 Å^−1^ have been chosen to ensure that all the enthalpy calculations are well converged. The phonon calculations have been carried out by using a supercell approach as implemented in the Phonopy code^53^. This method uses the forces obtained by the Hellmann-Feynman theorem calculated from the optimized 3 × 3 × 3 supercell. The Bader’s quantum theory of atoms in molecules (QTAIM) analysis^54^ was used for charge calculation.

Electron-phonon coupling (EPC) calculations have been performed using the pseudo-potential plane-wave method and density-functional perturbation theory^55^,^56^ as implemented in the Quantum-ESPRESSO package^57^. EPC calculation has been performed on 4 × 4 × 4 MP *q* meshes in the first Brillouin zone with a kinetic energy cutoff of 60 Ry. 16 × 16 × 16 MP *k* meshes are chosen to ensure *k*-point sampling convergence with Gaussians of 0.025 Ry, which approximates the zero-width limit in this calculation.

## Results and Discussions

### The high-pressure synthesis and structural information of CsI_n_

Structural predictions for CsI_n_ (*n* = 2 − 5) have been performed with CALYPSO methodology^32^,^33^,^34^,^35^ using simulation sizes with 1–4 formula per primitive cell under a series of pressure points (0, 50, 100, 150 and 200 GPa). The enthalpy of formation per atom is calculated using the following formula:


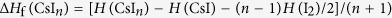


where Δ*H*_f_ is the enthalpy of formation per atom and *H* is the calculated enthalpy per chemical unit for each compound.

**Figure 1:**
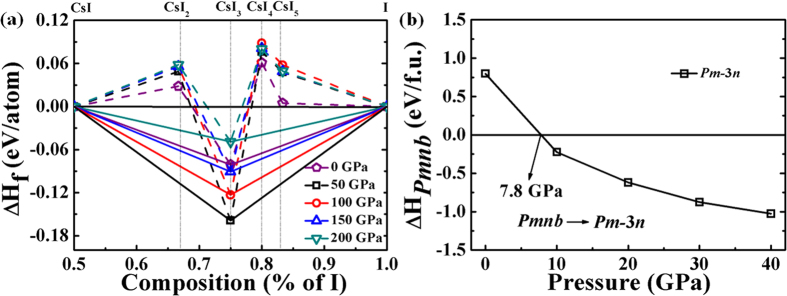
Enthalpy calculations of CsI_n_. (**a**) Enthalpies of formation of CsI_n_ under a range of pressures. Dotted lines connect data points, and solid lines denote the convex hull. (**b**) Enthalpy (related to the *Pmnb* phase) of *Pm*-3*n* structure for CsI_3_ as a function of pressures.

**Figure 2:**
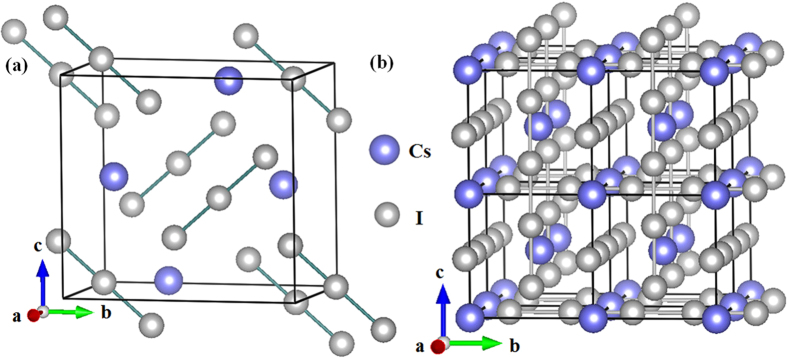
Crystal structures of CsI_3_. (**a**) CsI_3_ at 0 GPa with actual formula of Cs^+^[I_3_]^−^. (**b**) CsI_3_ at 10 GPa in a cubic *Pm*-3*n* structure.

From the convex hull shown in Fig. 1a (solid lines), only CsI_3_ with space group of *Pm*-3*n* is thermodynamically stable under high pressure. However, previous experimental work shows that CsI_4_ can be synthesized at ambient pressure, and a *P*2_1_*/a* phase with 4 formulas per primitive cell has been proposed by X-ray analysis^29^. The currently predicted *Pnma* phase is energetically much superior to the monoclinic *P*2_1_*/a* structure, while the formation enthalpy of *Pnma* is positive value and thus does not support its thermodynamic stability. We note that the experimentally synthesized CsI_4_ was heated in a closed vessel at 90 °C, and was then cooled slowly to 25 °C^29^. Therefore, the *P*2_1_*/a* CsI_4_ structure is a metastable phase. CsI_3_ is stable at ambient pressure and adopts an orthorhombic lattice with *Pmnb* symmetry (Fig. 2a). The optimized lattice parameter of *Pmnb* phase at 0 GPa is *a* = 7.2366 Å, *b* = 11.2631 Å and *c* = 10.4905 Å. At 0 GPa, this actually Cs^+^[I_3_]^−^ phase contains linear type I_3_^−^ ions with two I–I bonds length of 2.901 and 3.019 Å, which are similar to the experimental data of 2.820 and 3.100 Å^28^, respectively. Such typical hypervalent iodine moiety of orthorhombic *Pmnb* structure can be conventionally explained by traditional theory. For I_3_^−^ ion of *Pmnb* phase, the central iodine atom shares two electrons with two adjacent iodine atoms by forming one 3c-4e bond, and meanwhile it gets one electron from cesium atom to keep the other two iodine atoms following Lewis octet rule^23^. On the other hand, Pauling^58^ and Cartmell^59^ interpreted the hypervalence phenomenon of this ion in terms of the trigonal-bipyramidal orbitals of the central atom (*sp*^3^*d* hybridization). The central iodine atom has 10 electrons in valence shell which supports the conventional hypervalence phenomenon. The *Pmnb* structure remains stable up to 7.8 GPa, where undergoes a complicated first-order phase transition to cubic *Pm*-3*n* structure with volume collapse of 7.19% (Fig. 1b). The optimized lattice parameters of *Pm*-3*n* phase at 10 GPa is *a* = 6.2865 Å. Atoms occupy the Wyckoff 2*a* (0, 0, 0) for cesium and 6*d* (0, 0.5, 0.75) for iodine. Surprisingly, the enthalpies of *Pm*-3*n* phase at least up to 200 GPa from convex hull (Fig. 1a) show that *Pm*-3*n* phase tends to decompose as the increasing pressure.

**Figure 3:**
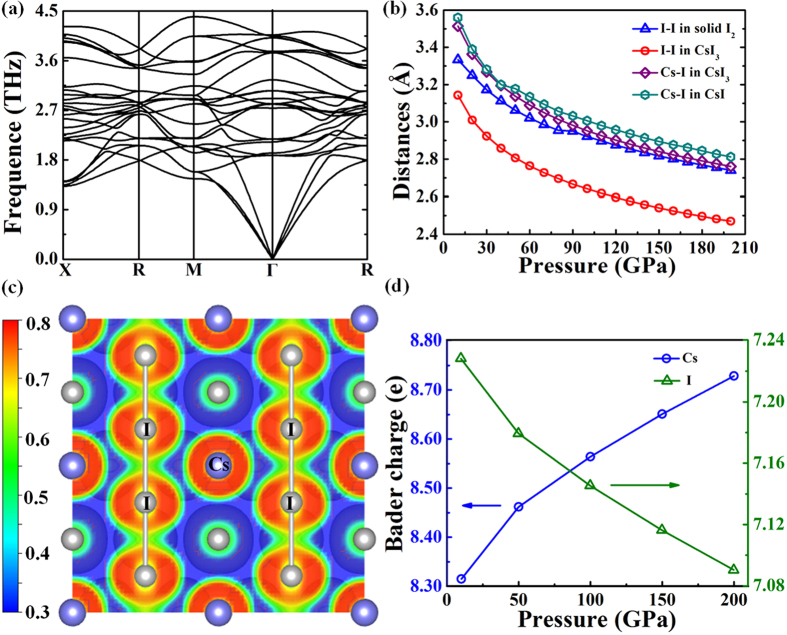
The lattice dynamics and bonding behavior analysis of *Pm*-3*n* CsI_3_. (**a**) Calculated phonon spectrum of *Pm*-3*n* CsI_3_ at 10 GPa. (**b**) Interatomic distances (between atoms shown in the structure directly underneath) in I_2_, CsI_3_ and CsI as a function of external pressure. (**c**) ELF of CsI_3_ at 150 GPa. (**d**) Calculated Bader charge of cesium and iodine in CsI_3_ as a function of external pressure.

Comparing with orthorhombic *Pmnb* structure, a striking feature of this modification is that all the iodine atoms form endless linear chains (Fig. 2b). And these endless chains just locate on the surface of the *Pm*-3*m* Cs lattice. The lattice dynamics calculations with no imaginary phonon frequencies support the dynamic stability of *Pm*-3*n* structure over the pressure range studied here (Fig. 3a). Remarkly, the endless chain structure has rarely been found in inorganic compounds before. One previous work shows that the B atoms of Li_2_B can form kinked chains along the c axis under high pressure^38^. However, such kinked chains of B is clearly different from iodine chains which are thoroughly linear without any tortuosity. The similar crystal structures with *Pnma* and *Pm*-3*n* space groups have been reported for NaCl_3_^31^. NaCl_3_ is stable in the *Pnma* containing almost linear asymmetric Cl_3_ groups at 20 to 48 GPa, and then transforms into a metallic *Pm*-3*n* structure. NaCl_3_ and CsI_3_ are clearly analogous compounds and even adopt the same structures, and Cl-Cl bonds in *Pm*-3*n* NaCl_3_ similar with our predicted I-I bonds by forming extended monatomic chains running along the three mutually perpendicular axes^31^. In addition, the Cs–I bond length in CsI_3_ is close to that in CsI as pressures change, indicating the forming of Cs–I ionic bond (Fig. 3b). And the I–I bond length in CsI_3_ is 2.764 Å at 60 GPa, significantly shorter than the shortest I–I distances of 3.021 Å in solid I_2_ (Fig. 3b), showing a stronger bonding character. The ELF of CsI_3_ at 150 GPa shows that the strongest electron localization area are located between iodine and iodine atoms (Fig. 3c), suggesting covalent bond forms between the iodine atoms. As we well know, pressure-induced molecular dissociation of solid I_2_ has been reported from theoretical or experimental studies, but iodine in *Pm*-3*n* phase can held together by strong chemical bonds at least until 200 GPa. We have calculated the charges using Bader’s quantum theory of atoms in molecules (QTAIM) analysis^54^ for CsI_3_ at high pressure. From Fig. 3d, the electrons devoted from cesium to iodine are gradually reduced as the increasing pressure, indicating a peculiar reverse electron donation from iodine to cesium, and thus naturally gives rise to suppress the strength of Cs–I bonds, which is responsible for tendency of the decomposition at higher pressure. Each iodine atom of CsI_3_ gets two shared electrons from the nearest-neighbor iodine and averagely 0.3 electrons from cesium atom, and frankly the valence shell of iodine has 9.3 electrons with hypervalence character. Generally, several hypervalent compounds such as hypervalent P, S, I and Xe in PCl_5_, SF_6_, IF_7_ and XeF_4_ always have integral electrons in valence shell of 10, 12, 14 and 12, respectively^60^. For conventional hypervalent iodine compounds, only one central atom possesses hypervalence property which being surrounded by other several ligands. While in the current ionic CsI_3_ compound, all the iodine atoms possess such character, which broadens conventional understanding of hypervalent iodine. Therefore, high pressure induces the attractive hypervalent phenomenon in CsI_3_ iodine with specific endless linear chain structure.

**Figure 4:**
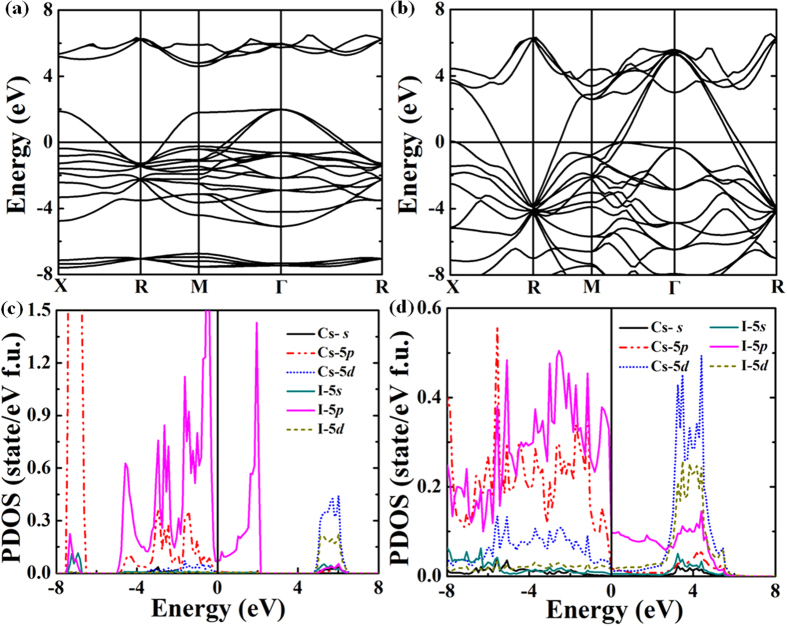
The electronic property analysis of *Pm*-3*n* CsI_3_. Calculated electronic band plot along high-symmetry directions of *Pm*-3*n* CsI_3_ at 10 GPa (**a**) and 150 GPa (**b**). Electronic density of states for *Pm*-3*n* CsI_3_ at 10 GPa (**c**) and 150 GPa (**d**).

At ambient pressure, the prototypical ionic crystal of CsI_3_ is an insulator. Interestingly, CsI_3_ becomes metallic after the first-order phase transition at 7.8 GPa. The calculated electronic band structures of *Pm*-3*n* phase at 10 GPa show it is metallic with several bands crossing Fermi level (Fig. 4a). Pressure induces CsI_3_ to exhibit increasingly shorter interatomic distances, which is accompanied by an increase in the bandwidth, especially those near the Fermi-surface, thus leading to the 5*p* bands of iodine expand along conductive band direction (Fig. 4b). Furthermore, the calculated projected density of states (PDOS) of CsI_3_ show that the 5*d* states of Cs have more electronic occupation as the pressure increasing (Fig. 4c,d).

It should be mentioned that the superconducting behaviors in CsI have been extensively explored through experimental measurement and theoretical calculations^12^,^13^,^14^. It is suggested that the formation of several electrons and hole Fermi-surface pockets in CsI is due to a dramatic increase of the electron donation from I^−^ to Cs^+^, thus leading to more electrons to be involved in the electron-phonon coupling which is responsible for superconductivity and the larger EPC potential, thereby contributing to the increase in *T*_*C*_^12^. Therefore, it is intriguing to determine whether *Pm*-3*n* CsI_3_ will possess superconducting property under high pressure.

**Figure 5:**
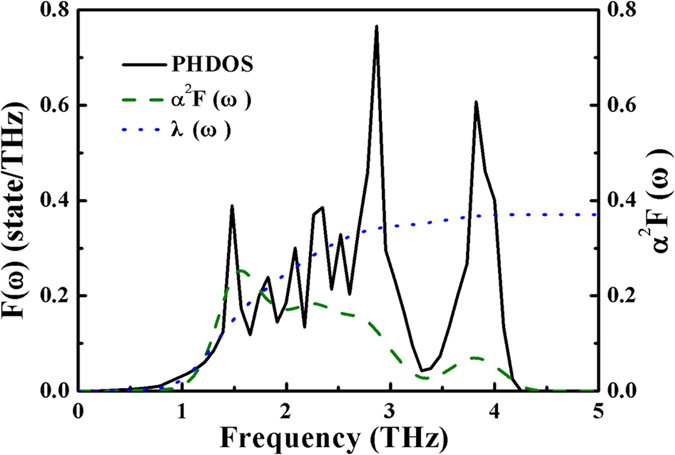
The electron-phonon coupling calculations of *Pm*-3*n* CsI_3_. The calculated phonon density of states (solid line), Eliashberg spectral function *a*^2^*F* (*ω*) (dashed line), and the electron-phonon integral (dotted line) of *Pm*-3*n* CsI_3_ at 10 GPa.

Figure 5 shows the phonon density of states (PHDOS) and Eliashberg spectral function *α*^2^*F* (*ω*)^61^ for *Pm*-3*n* CsI_3_ at 10 GPa. It is found that *α*^2^*F* (*ω*) contains two parts of which the low and high frequency area are localized within a narrow vibrational region of 0.8–3.4 THz and 3.4–4.2 THz, respectively. The integrated EPC λ of low frequency *α*^2^*F* (*ω*) are 0.353 constituting 95.66% of the total λ 0.369, and the other EPC λ in high frequency area is 0.016 accounts 4.34% of total λ. With increasing pressure to 150 GPa, λ was reduced to be 0.074. The superconducting critical temperature *T*_*C*_ can be estimated from the Allen-Dynes modified McMillan equation^62^ 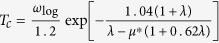, where *ω*_log_ is the logarithmic average frequency calculated directly from the phonon spectrum and μ^*^ is the Coulomb pseudopotential. *ω*_log_ is obtained to be 97.33 K at 10 GPa and 322.96 K at 150 GPa. Using a μ^*^ of 0.1, the estimated *T*_*C*_ is 0.15 K at 10 GPa but it disappears at 150 GPa. It is noteworthy that the estimated *T*_*C*_ in CsI_3_ is noticeably larger than the value in CsI of 0.03 K, and the pressure is significantly lower than that of CsI (180 GPa).

## Conclusions

In summary, we have systematically investigated compounds CsI_n_ up to 200 GPa using an effective CALYPSO algorithm. Strikingly, CsI_3_ with space group of *Pm*-3*n* is thermodynamic stability under high pressure. Within the cubic *Pm*-3*n* CsI_3_ phase, iodine atoms arranging into several endless linear chains show an attractive hypervalence phenomenon under high pressure, which is in sharp contrast to the conventional understanding. We further discovered that *Pm*-3*n* CsI_3_ is a metallic phase with several energy bands crossing Fermi-surface, and the pressure creates a peculiar reverse electron donation from iodine to cesium. The electron-phonon coupling calculations have proposed superconductive potential of the metallic *Pm*-3*n* CsI_3_ at 10 GPa, which are analogous to the known CsI^12^. This work has wide implications for other inorganic compounds that likely harbor similar high-pressure behavior, and the significance for synthetic chemistry is highlighted.

## Additional Information

**How to cite this article**: Wei, S. *et al*. Hypervalent Iodine with Linear Chain at High Pressure. *Sci. Rep*. **5**, 14393; doi: 10.1038/srep14393 (2015).
